# Experiences of men who have sex with men in accessing HIV counselling and testing in Mdantsane Township, South Africa

**DOI:** 10.4102/phcfm.v16i1.4632

**Published:** 2024-11-18

**Authors:** Lusindiso Holiday, Nelisiwe Khuzwayo

**Affiliations:** 1 Department of Public Health Medicine, College of Health Sciences, University of KwaZulu-Natal, Durban, South Africa

**Keywords:** HIV counselling and testing, men who have sex with men, stigma, discrimination, primary health care, sexual minorities, South Africa

## Abstract

**Background:**

Despite scientific evidence of a rapid increase in the number of men who have sex with men (MSM) who test positive for HIV, many African states continued to ignore and exclude MSM in healthcare planning and provision, resulting in their reluctance and refusal to access healthcare services.

**Aim:**

This study aimed to explore, the lived experiences of MSM in accessing HIV Counselling and Testing services within the primary health care system in a township in South Africa.

**Setting:**

Mdantsane Township, outside East London, Eastern Cape province.

**Methods:**

This qualitative study was placed within the interpretive paradigm and employed a descriptive phenomenological research design. A non-probability purposive sampling technique was used to recruit participants. The study was inductive and utilised semi-structured in-depth interviews and an interview guide to collect data. A descriptive phenomenological data analysis method was employed to process and analyse the data.

**Results:**

Five main themes emerged from the study, namely, (1) individual factors related to MSM’s experiences of nearby healthcare centres providing HCT; (2) interpersonal factors related to their experiences inside the clinic; (3) institutional factors related to lack of sensitisation; (4) community factors related to the role of public-private partnerships in improving healthcare services for MSM; and (5) public policy factors related to the role of government in ensuring accessibility of HCT policy to MSM.

**Conclusion:**

Access to HCT services for MSM in Mdantsane was levels and there is an urgent need influenced by a myriad of complex factors at different socio-ecological levels, and there was an urgent need for tailormade interventions at all these socio-ecological levels to address challenges.

**Contribution:**

This study contributes to scientific knowledge by bringing forth the voices of sexual minorities regarding the quality of healthcare service provision they expect to receive from their local clinics.

## Introduction

Globally, there were an estimated 38.4 million people living with HIV at the end of 2021,^1^ an increase of about 501 000 new infections from the last recorded statistics of nearly 37.9 million people who were living with HIV in 2018.^2^ Moreover, an estimated 20.6 million people are living with HIV in Eastern and Southern Africa, which works out to more than half (54%) of all people living with HIV in the world.^2^ Furthermore, South Africa is believed to have the highest number of people living with HIV globally, with an estimated total number of 8.45 million HIV-positive people in 2022.^3^ A report preceding the 2022 report was released in 2019, and it estimated that approximately 7.6 million people were living with HIV in South Africa at the end of 2019;^4,5^ a slight decrease of about 100 000 from the 2018 report released before that which gave an estimation of 7.7 million confirmed HIV-positive cases around 2018.^2^ Of that global total of 38.4 million HIV people in 2021, key populations (KP) groups (inclusive of men who have sex with men [MSM]) and their sexual partners accounted for over 62% of all new HIV infections globally among the age group of 15–49 years.^6^ Meanwhile, in 2016, the same KPs and their partners in other regions of the world outside of sub-Saharan Africa accounted for 80% of new HIV infections^7^, whereas in sub-Saharan Africa, KPs and their sexual partners had accounted for 25% of new HIV infections in 2016.^8,9^

Amid the above-stated statistics displaying high HIV prevalence, there is the issue of access to HIV Counselling and Testing (HCT) for MSM and other sexual minorities, which this study seeks to investigate about MSM’s lived experiences when seeking the service at their local primary health care (PHC) centres. HIV Counselling and Testing intake for MSM in Africa is reported to be still very low compared to the general population. This, therefore, creates a need for strategic and focussed targeting of HCT education and services for MSM to ensure that they and other members of the critical populations are reached and can access all HCT and support services.^10^

Globally, various structural factors limit the ability of MSM to access essential, appropriate and acceptable HIV prevention and treatment services.^11^ Some of those structural factors include stigma and discrimination, homophobia, a lack of confidentiality and anonymity, a lack of awareness, unemployment and poverty and illiteracy.^12^ Duby et al. further opine that laws that criminalise same-sex relationships in some African states marginalise MSM and perpetuate their exclusion from their communities and essential support services.^11^ Men who have sex with men commonly experience disapproval, rejection and suboptimal services in healthcare settings, to the point of their exclusion from the formal health system altogether.^11^

This study relied on the Socio-ecological Model for Health Promotion (SEM-HP) to provide a theoretical framework for understanding health-seeking behaviour regarding access to HCT services for MSM. Max et al. defined the SEM-HP as a theory-based framework for understanding, exploring and addressing the social determinants of health at many levels.^13^ They stated that ‘ecological’ means multiple levels beyond the individual. Thus, the SEM-HP demonstrates that behaviour results from the knowledge, values and attitudes of individuals’ social influences, including the people with whom they associate, the organisations to which they belong and the communities in which they live.^13^ They further stated that the SEM-HP encourages people to move beyond a focus on individual behaviour and towards an understanding of the wide range of factors that influence health outcomes. This approach supports the building of collaborations beyond just the accessing of HCT services for MSM (for example) to the total management of healthcare services from the policy level right down to the individual and/or intrapersonal level where accessing such healthcare services takes place.^13^

The aim of this study was to explore, describe and document the lived experiences of MSM in accessing HCT services within the primary care system in Mdantsane. Meanwhile, the objectives the study sought to achieve included identifying and describing the lived experiences of MSM who access HCT services through the primary care system in Mdantsane, establishing what it felt like to be an MSM that seeks HCT services at a PHC centre in Mdantsane; establishing what the stories MSM construct out of their lived experiences of accessing HCT at PHC centres mean to them; determining how the articulation of these lived experiences empower MSM; describing the MSM’s understanding of the experiences they encounter when accessing HCT at the PHC centres and establishing how these experiences encountered at the PHC centres affect the way MSM perceive HCT.

## Research methods and design

### Study design

This was a qualitative study, and it was placed within the interpretive paradigm. The study employed a descriptive phenomenological research design. Phenomenological research aims to describe experiences as they are lived; in other words, the ‘lived experiences’.^14^ The phenomenological research method was chosen and utilised in this study mainly because of its interest in investigating, describing and analysing the lived personal experiences of participants concerning a particular observed phenomenon: MSM’s accessing of HCT services within the primary care system in South Africa, in this case. The primary researcher for this study believes that the combination and complementing nature of the interpretive paradigm, the qualitative research method and the phenomenological research design is best suited to describing the lived experiences of MSM who access HCT in their local clinics as they are embedded and embodied in the words that represent their feelings and experiences.

### Setting

This study was conducted at Mdantsane Township, outside East London, in the Buffalo City Metropolitan Municipality, Eastern Cape province, South Africa.

### Study population and sampling strategy

The target population for this study was MSM between the ages of 18 and 55 years, living within the township of Mdantsane. This age bracket was strategically chosen because people within it were most likely to be sexually active and would most likely want to access HCT services if they were not already accessing them. A non-probability purposive sampling technique was utilised to select a sample. The saturation principle was applied to determine the study’s sample size. The sample size of the study consisted of 10 men who were having sex with other men from Mdantsane Township.

### Data collection

This study utilised semi-structured in-depth interviews and an interview guide to collect data from 10 participants who identified as MSM. Each participant was interviewed in the comfort of their home. Questions asked in the interviews required the participants to share their lived experiences of visiting local clinics to seek HCT, the kind of reception they received from the clinic staff, questions they were asked by healthcare workers, the step-by-step process that was followed to offer them HCT, what they liked or disliked about the service they received from the healthcare workers, the kind of and usefulness of the information that was shared with them after they were given their HIV results, whether they would go to the same local clinic again after their experience at that clinic and the changes and/or suggestions they would like to propose to healthcare workers to improve quality of the service rendered at the clinic.

Mdantsane Township is a predominantly Xhosa settlement area; as such, the isiXhosa language was used throughout the interviews. Consent was sought from all 10 participants before each interview was conducted. The interviews were audio-recorded with permission, and each lasted about 1 h and a half. Each participant’s interview was assigned a code to protect the participant’s identity.

### Data analysis

A descriptive phenomenological data analysis method was utilised to analyse data, and it followed the seven steps of the Colaizzi’s descriptive phenomenological method, which is mostly utilised to analyse data within health sciences research. These steps were familiarisation, identifying significant statements, formulating meanings, clustering themes developing an exhaustive description, producing the fundamental structure and seeking verification of the underlying structure.^15^ After audio-recording the interviews, followed by a verbatim transcription of the interviews and later the translation of the transcribed data from isiXhosa language to the English language, the principal investigator started familiarising himself with the data by reading and re-reading all the transcripts individually to get a general sense of the content. The next step entailed identifying significant statements that relate to the phenomenon under study and extracting them from transcripts. From significant statements, meanings were then formulated. Formulating meanings entails recognising meanings that are close to the study by bracketing the researcher’s knowledge of the formulated meaning. Clustering of themes followed and it involved organising the formulated meanings into clusters of themes. Following the clustering, development of exhaustive descriptions took place. This was achieved by integrating the findings (including all themes that were developed) into exhaustive descriptions. The above step was followed by the production of the fundamental structure, which entails the description of the fundamental structure of the phenomenon by ensuring that reports covered all the essentials. In the last step, verification of the underlying structure was achieved by validating the findings with study participants (taking the fundamental structure statements to all study participants for confirmation). Open coding was utilised to analyse the data. Additionally, the Consolidated Criteria for Reporting Qualitative research (COREQ) checklist was also utilised to maintain the quality of the data.

Lastly, this study was conducted as a requirement towards the fulfilment of an academic programme (Master’s degree); an academic lecturer who supervised the study also played a role of being a co-coder for the study as required by the institution.

### Trustworthiness

Trustworthiness is a method by which researchers can persuade and convince themselves and readers that their research findings are worthy of attention.^16^ For a study to be accepted as trustworthy, qualitative researchers must demonstrate that data analysis has been conducted in a precise, consistent and exhaustive manner through recording, systematising and disclosing the analysis methods with enough detail to enable the reader to determine whether the process is credible.^16^ Trustworthiness in this study was achieved by ensuring that the study’s credibility, transferability, dependability and confirmability^17^ were observed and achieved throughout the study process. The principal investigator kept a reflective diary to ensure that objectivity and neutrality were maintained throughout the process. Interview recordings were translated verbatim to enhance the authenticity of the study. Prolonged engagement with the data sets, data triangulation and a co-coder in form of an academic research supervisor were also involved in order to achieve credibility. The research supervisor was not involved in data collection and she played a role of an independent co-coder. A 2-quire counterbook containing all interview transcripts was kept and locked at the principal investigator’s house in order to ensure that a record and an audit trail were preserved so that any other researcher could be able to access the scripts on request and follow up on the study. Principal investigator ensured dependability by referring to the recordings many times, coding and re-coding and sharing the themes and categories with the research supervisor for reflection and comparison.

### Ethical considerations

Ethical approval for this study was obtained from the University of KwaZulu-Natal (UKZN) Research and Ethics Committee (No. HSSREC/00001502/2020). Lastly, a letter was written and sent to the non-governmental organisation (NGO) that the principal investigator identified to ask for permission to recruit and conduct interviews with some of the MSM they have worked with in the past. All recruited participants were given a participant information sheet and an informed consent form. The forms were read and explained to them before they could sign them. Participants were given an option to exit the interview process anytime during the interview should they feel the need to discontinue.

The study was guided by the following ethical principles and guiding documents, which helped to direct and regulate the ethical conduct of the principal researcher and the study subjects throughout the duration of the study:

### Informed consent

The participants signed informed consent before the interviews, and its contents were read to each participant individually to ensure they understood it. The consent form was in isiXhosa as the local language and was also made available in English for whoever may feel comfortable with the English language version. Moreover, each participant had to sign a consent form, which certified that they agreed with its contents and were willing to participate in the study. The recruitment process was done with the NGO’s assistance, which helped identify the 10 participants.

### Confidentiality and privacy

Anonymity or privacy was achieved by using coding instead of the names of the participants. The names of the participants were not written in any of the interview transcripts, nor was there anything written down that would make someone easily link the completed interview script to any of the participants. Confidentiality was maintained by not involving anybody else during the data collection period. The principal researcher was the only one to meet up with participants and the only one to interview them at their private places of choice.

## Results

### Socio-demographic characteristics

Interviews were conducted with a total of 10 men who were having sex with other men. All these men were from the amaXhosa tribe residing in the township of Mdantsane. These men were ranging from the age of 23 to 34 years. Most of these men identified themselves as gay, while a few of them identified as bisexual and queer, respectively. All these men had some level of education, with some having matric as their highest education and others university graduates and drop-outs. While some of these men were working, some were unemployed, some were self-employed and some of them were still students.

Table 1 shows the socio-demographic characteristics of the respondents.

**TABLE 1:** Socio-demographic characteristics of the participants.

Participant code	Age (in years)	Sex at birth	Sexual orientation	Ethnicity
A09	34	Male	Gay	Xhosa
B09	24	Male	Bisexual	Xhosa
C11	27	Male	Gay	Xhosa
D12	29	Male	Gay	Xhosa
E13	32	Male	Gay	Xhosa
F13	23	Male	Queer	Xhosa
G14	25	Male	Bisexual	Xhosa
H14	32	Male	Gay	Xhosa
I18	26	Male	Gay	Xhosa
J19	27	Male	Gay	Xhosa

## Themes emerging from the study

Upon analysing the study data, five themes emerged. Emerged themes included:

1. individual or intrapersonal level factors;
2. interpersonal level factors;
3. institutional level factors;
4. community level factors; and
5. public policy level factors.

### Theme 1: Individual or intrapersonal level

#### Sub-theme 1.1: Knowledge of HIV counselling and testing and healthcare centres providing HIV counselling and testing services

In-depth interviews revealed that MSM’s knowledge of what HCT was and what the HCT process entailed gave them the confidence to want to access HCT. In addition, the in-depth interviews revealed that the knowledge of the benefits that come with knowing one’s HIV status also motivated MSM to go and test for HIV. Furthermore, MSM whose HCT results came back negative were highly likely to go back and test again:

‘In my work, I’m used to working with clinics and NGOs like ANOVA and Engage Men’s Health. So, I know what gets done there when they test you and the importance of knowing my status. Hence, I always test whenever I get a chance.’ (Participant A09, 34 years, Male)

‘… I am also not scared to go and test whenever I am at the clinic because I know my status. I am negative.’ (Participant B09, 21 years, Male)

#### Sub-theme 1.2: The influence of proximity and convenience in men who have sex with men’s personal decision to access HIV counselling and testing

Data from the in-depth interviews also revealed that the proximity of healthcare centres that provide HCT services gave MSM convenience and easy access to healthcare centres. The convenience was a positive contributing factor in most MSM’s decision to visit a clinic, and the closer was the clinic to their homes, the more likely they were to visit that particular clinic. Proximity to the clinic allowed them to access the clinic anytime they wanted. Sometimes, even if they were lazy to go to the clinic, the fact that the clinic was nearby motivated them enough to go. One of the participants shared that he chose to visit his clinic of choice because:

‘“It was closer home” and “It’s convenient”.’ (Participant A09, 34 years, Male)

Additionally, knowing that there was a nearby healthcare centre providing HCT was another motivating factor for MSM to go and test for HIV:

‘Where I was staying before the clinic was just around the corner. I would go to test often. But where I am staying now the clinic is a bit far, and I am lazy to go.’ (Participant B09, 21 years, Male)

### Theme 2: Interpersonal level

#### Sub-theme 2.1: Personal experiences of men who have sex with men inside a clinic consultation room

This sub-theme gave insights into the actual lived experiences of MSM inside the consultation rooms of their local clinics. The sub-theme further produced other sub-themes, and some of those sub-themes that emerged out of this sub-theme include the following: (1) Healthcare workers’ inability to understand and accept the existence of MSM, (2) segregation of clients inside the clinics, (3) Inconsistencies in the process of administering HCT, (4) healthcare workers’ religious beliefs as a prohibiting factor to accessing PHC, (5) healthcare workers’ choice of words as a deterring factor to accessing HCT for MSM.

**Sub-theme 2.1.1: Healthcare workers’ inability to understand and accept the existence of men who have sex with men:** Analysed data revealed that most participants felt nurses were not ready to accept that men were having sex with other men. These participants stated that many of the nurses saw the phenomenon of men who have sex with other men as obscene, taboo and unacceptable. One participant reported that:

‘Nurses are not yet ready to accept that men have sex with other men. They see it as obscenity.’ (Participant D12, 28 years, Male)

**Sub-theme 2.1.2: Segregation of clients inside the clinics:** Other participants shared that they did not like the fact that people were divided into groups inside the clinics according to their clinical needs or what they came to do at the clinic. Participants shared that clients who came to do HCT were put on one side, separate from those who came for other clinical services. One participant shared:

‘No. I would never go back there. I hate the dividing of people. The experience I had was not lekker (nice in Afrikaans) at all. It was not nice because, first thing, there are people who are put aside. Everyone knows that those people are there to test for HIV. And then now, as you leave the clinic, people are looking at you because they want to see your facial expression. And then, the minute they see your facial expression, they know the outcome of your results. You see?’ (Participant E13, 36 years, Male)

**Sub-theme 2.1.3: Inconsistencies in the process of administering HIV counselling and testing:** The data from the in-depth interviews further revealed that the flouting of processes and procedures of HCT was rife within the primary care centres in Mdantsane Township. Healthcare workers need to follow the correct procedures and processes when conducting HCT. Men who have sex with men reported that healthcare workers conducted tests whichever way they felt like it. One participant indicated that the nurse testing him needed to follow the standard process of testing he was used to, the *pre-test counselling*, the *test* and the *post-test counselling* (in that order). The participant stated that the nurse did not even counsel him. The nurse just jumped straight into testing. The participant highlighted this by stating that counselling was never part of his HCT experience. The participant shared:

‘I don’t remember getting counselled. I don’t know if they don’t offer counselling or it was due to lack of staff, but counselling was never a part of it.’ (Participant A09, 34 years, Male)

**Sub-theme 2.1.4: Healthcare workers’ religious beliefs as a prohibiting factor to accessing primary health care:** Most participants also shared that some of their challenges at the clinics were that some nurses used their religious beliefs to discriminate against people who have sex with people of the same sex. Nurses who subscribed to the Christian religion were perceived as the main culprits of this behaviour. Participants reported that nurses who attended to them preached religious scriptures to them from the onset without checking their religious views first. The participant stated that the nurse who attended to them once discriminated against them because of her religion and beliefs that conflicted with her professional mandate, which eventually prevented her from executing her professional duties and responsibilities:

‘She preached at me about the Bible. The problem is that they drag their religion and beliefs to work, forgetting that they are supposed to leave their religion at the gate when they enter the clinic premises. I don’t ever want to go to that clinic and get the same experience I got when I was there.’ (Participant D12, 28 years, Male)

**Sub-theme 2.1.5: Healthcare workers’ choice of words as a deterring factor to accessing HIV counselling and testing for men who have sex with men:** The language and choice of words that some healthcare workers used when communicating and interacting with MSM was seen as a deterrence to seeking healthcare services by some MSM. Most participants shared that the tone used by other nurses when asking them questions sounded wrong, accusing and lacked professionalism. One participant shared that starting the conversation with ‘How can I help you?’ or ‘What can I do for you today?’ would have sounded much better and inviting to him than ‘Do you want to test for HIV?’ that he received from one nurse at his local clinic. Following are some of the ‘unacceptable statements’ healthcare workers began their HCT sessions with:

‘He asked me if I wanted to do the HIV test. I said yes.’ (Participant B09, 21 years, Male)

‘He asked what was the reason for me to want to do the test. I said I wanted to know if I was HIV negative or positive.’ (Participant F13, 26 years, Male)

### Theme 3: Institutional or organisational level

#### Sub-theme 3.1: Standing in long queues at the clinic

Most participants stated that one of the worst experiences when visiting their local clinics to access HCT was standing in long queues the entire day before they got to be attended. Participants shared that they usually would have to wake up very early when going to the clinic so that they could arrive around 06:00, only to be attended when the clinic was about to close around 4:30 pm:

‘There are always long queues at that clinic. You will find out that if you go to that clinic, you will arrive at 6 am and leave at 4:30 pm when they close the clinic. The nurses there drag their feet, and they take forever to help people.’ (Participant C11, 25 years, Male)

#### Sub-theme 3.2: Lack of privacy, professionalism, and confidentiality

According to the analysed data, the lack of privacy, the lack of professionalism and the lack of confidentiality served as deterrents to most MSM accessing HCT at their local clinics. Participants shared how other clinic workers would walk in and out of the consultation room while they were being attended to. Other participants also narrated how the nurses would call other nurses to the consultation room to hear about the client’s sex life and sexual behaviour or preferences. One participant gave an account of how embarrassing that experience was, and as such, he felt that the clinic was not a confidential space:

‘It’s not a confidential space. Other clinic workers can get in and out of the counselling room to take some stuff while you are there. The nurse could call other nurses to hear what you say about your sex life. Then they will make fun of you and your sexual preference. It’s embarrassing.’ (Participant B09, 21 years, Male)

#### Sub-theme 3.3: Lack of sensitisation, education and understanding of men who have sex with men

The lack of sensitisation, the lack of human sexuality education and the lack of understanding of MSM on the part of healthcare workers emerged widely on many participants’ in-depth interviews. Most participants shared that healthcare workers needed to be educated and sensitised about human sexuality, the diversity among human beings and how to better handle and embrace these differences and diversities in sexuality. One participant shared that:

‘That clinic needs to do a clinic staff training to sensitise the staff about the queer community. They must not judge a person because he is queer and deny him the service he wanted and preach to him instead. They must do sensitisation training. That could also help us feel like we belong to that clinic when we visit.’ (Participant D12, 28 years, Male)

### Theme 4: Community level

#### Sub-theme 4.1: Public-private partnerships for improved healthcare services

Analysed data also revealed that partnerships between government clinics and NGOs emerged as a sub-theme and a suggestion that could improve service delivery within the PHC system in the community of Mdantsane Township. Some participants shared that they were no longer going to their local clinics for HCT and other sexual health services because the service was poor in those clinics. Instead, they were now going to alternative healthcare providers such as NGOs who treated them with utmost respect and professionalism. One participant shared the following:

‘I think, honestly speaking, the partnership between the clinics and ANOVA, Beyond Zero and other like-organisations is important because those NGOs give us a better service.’ (Participant J19, 30 years, Male)

### Theme 5: Public policy level

#### Sub-theme 5.1: The role of government in ensuring accessibility of HIV counselling and testing policy to men who have sex with men community members

Under the theme of ‘public policy level’, data analysed from the in-depth interviews (IDIs) revealed the views of MSM regarding the role of government as a primary custodian of HCT policies and all other relevant health policies that govern the administration of health services in the Mdantsane Township area. The general view and feeling from most participants were that most MSM did not know of any policies in place that were meant to address the issue of access to HCT for MSM. Participants stated that when the government comes up with a policy to address the provision of healthcare services for MSM, it should embark on educating all healthcare workers about such policy and further ensure that the policy is enforced on healthcare workers to implement it and make it work for the populations it was intended to serve. One participant shared that:

‘I touched on educating healthcare workers and forcing them to utilise the policies because if the policies are just sitting there and not being utilised, they are as good as non-existent.’ (Participant A09, 34 years, Male)

Data also revealed that participants suggested that as and when these policies become available, they should be made available and visible to patients and community members by putting them up on clinic walls and noticeboards so that everyone who visits the clinic can see familiarise themselves with them. A participant shared that:

‘The policies must be put up on the wall or notice board so that I could also read them and know what kind of service to expect from the clinic.’ (Participant I18, 29 years, Male)

Analysed data from IDIs further revealed that participants wanted the government to ensure that available policies on providing healthcare services to MSM community members should be disseminated through different media platforms. According to the participants, this would ensure that MSM and other community members could access them and familiarise themselves with their content. Another participant shared that:

‘Government should post these policies on social media to be easily accessible to MSM and the general population. On WhatsApp, YouTube, Facebook, etc.’ (Participant F13, 26 years, Male)

## Discussion

This study aimed to investigate the lived experiences of MSM who access HCT services within the PHC system in Mdantsane Township. The findings in this study confirmed that MSM were experiencing many challenges when visiting their local clinics to access HCT services. Some of the experiences MSM encountered within their local clinics included discrimination, stigma, prejudice, a lack of social support,^17^ long distances, costs associated with travelling, standing in long queues, perceived quality of care, patient segregation, homophobia and confidentiality issues that discouraged the use of services rendered in those clinics.^18^

Proximity of healthcare centres that provide HCT services gave MSM convenience and that convenience was a positive contributing factor in their decision to whether to visit a clinic or not. Men who have sex with men were likely to visit a clinic within their reach, preferably the one they could walk in without transportation. Although there are areas in which people travel long distances to access health services, the National Department of Health of the South African government, through a PHC approach, ensures the accessibility and affordability of health services for the citizens. The findings of this study align with another study that reported the importance of taking services closer to where people live and work.^19^

Participants in the study viewed their experiences when accessing HCT within the clinics as unpleasant because of stigma and the attitudes of the health workers towards MSM. The negative attitudes of the health workers towards patients have long been identified, especially in HIV/AIDS studies and youth risk behaviours.^20^ These findings mirror the study conducted by Lorenc et al., whose study findings reported stigma as a significant barrier among MSM globally.

Patient segregation was a serious problem within the local clinics in Mdantsane as it exposed clients who came to do HCT and made it evident for everyone to see that they had come to test for HIV. This raised questions about the breach of confidentiality as many MSM felt that the segregation revealed their medical conditions to everyone and undermined their privacy or lack thereof. Those clients who were seen in the queue for HCT were likely to encounter prejudice and stigma related to ‘testing for HIV’. This finding aligns with other findings in a study conducted in KwaZulu-Natal, South Africa, which highlighted the segregation of patients in clinics as a barrier to linkage to care.^21^ Another study stated that participants did not want to be seen in a queue going to collect antiretrovirals therapy (ARVs) and that the separation from other clinic services resulted in them constantly feeling as being ‘othered’ within the facility.^22^

Men who have sex with men also reported inconsistencies in the administration of HCT within their local clinics. These inconsistencies included mainly the flouting of HCT processes and procedures. According to the participants, the process differed from the normal HCT procedure, a standard testing process as they knew it. For instance, participants shared that there was no ‘*pre-test counselling*, actual *test* and *post-test counselling*’ (in that order) as they were used to it. This inconsistency in the administration of HCT leaves one with questions regarding the preparedness and readiness of those MSM who are receiving the HCT from those nurses to deal with the test results. Previous research on HCT states that the main idea of pre-and post-test counselling is to ensure that individuals are guided throughout the HCT process to know what to expect and what to do when they receive a negative or positive HIV result.^23^ The KwaZulu-Natal Department of Health emphasises the importance of following the proper HCT process, describing the process as an opportunity for an individual who will undergo HIV testing to make informed decisions about whether to be tested for HIV.^24^ If clients are not being adequately counselled as the MSM participants in this study claim (e.g. the omission of pre-test and post-test counselling), how will they make informed decisions about their anticipated HCT process and the test results? According to The Global Forum on MSM & HIV,^24^ an appropriate and correct HCT process includes pre-test counselling that outlines the testing process, a risk behaviour assessment, the informed consent of each participant, the actual administration of the test and post-test counselling based on the test results.^25^

Nurses who are Christian were perceived as problematic within the PHC centres. Men who have sex with men accuse these nurses of sharing bible scriptures that seemingly condemn relationships between people of the same sex. The nurse’s religious beliefs conflicted with their professional mandate, eventually preventing them from executing their professional duties and responsibilities as expected. An existing study conducted in South Africa in 2011 supports the above reports by stating that mistreatment of MSM, gay men and other KPs such as MSM, sex workers (SW) and people who inject drugs (PWID) (which could sometimes emanate from nurses’ religious beliefs) by healthcare workers was a significant barrier to accessing health services.^11^

The choice of words used by some healthcare workers when communicating and interacting with MSM was reported as inappropriate and offensive. This was displayed in the tone used by other nurses when asking MSM questions about their sexual behaviour. Men who have sex with men felt the nurses’ questions were confrontational, accusing and unprofessional. Previous studies on experiences of HCT among MSM corroborated this finding by stating the ‘disparaging things’ said by some nurses about MSM and other people diagnosed with HIV. Some nurses have been recorded shaming MSM and people diagnosed with HIV by referring to them as ‘sexually promiscuous’.^26^

Men who have sex with men reported that long waits at local clinics for HCT were among their worst experiences, often arriving as early as 06:00 but not being seen until after 16:00. This discouraging experience led many to avoid returning, feeling it was a waste of time. They also criticised healthcare workers for being slow and unprofessional. These findings align with another study where MSM negatively reviewed clinics because of long waiting times, excessive talking by nurses and a lack of professionalism among staff.^27^

Participants expressed the need for the government to expeditiously develop tailor-made health policies that would push for the prioritisation of access to healthcare for MSM and other sexual minorities. Most MSM interviewed in the study expressed that the government should develop health and HCT policies that were inclusive and embraced diversity in sexualities, and that the government had to ensure that the policies were consistently implemented across all local clinics in all communities. Also, the government needed to ensure that the policies worked for and served the needs and interests of MSM and other marginalised groups they were developed for. Through the Department of Health, the government should also ensure that healthcare workers are well versed with the policies, enforce that healthcare workers implement them as they were adopted and published and not be selective in implementing them. Moreover, participants expressed that the government must not end up developing these MSM-friendly policies but must ensure they are made available and easily accessible to MSM and the general community. They emphasised the need to have these policies accessible to the general community and be followed up by community education programmes that will help to educate the communities in which MSM live and come from. By so doing, the communities would also be sensitised about the existence of MSM and acceptable ways to deal, interact and co-exist. Lastly, interviewed MSM emphasised the publication and dissemination of the policies so that they can be accessible to the service end-users, and they suggested that the government should make use of the latest mass media technologies such as WhatsApp, Facebook and YouTube, which most people utilise and have daily access to educate people about these policies. These findings align with similar findings on another policy study conducted by the African Men for Sexual Health and Rights (AMSHeR) and the Health Policy Project (HPP) in sub-Saharan Africa. The study opined that laws and other discriminatory practices and policies force an already marginalised community further underground, threatening their human rights, limiting their access to health services and increasing their risk of sexually transmitted infections (STIs), HIV, mental health conditions, poor nutrition and other health-related disparities.^28^ According to the study, governments and advocacy organisations should work alongside MSM communities to support MSM through free HIV and STI testing, psychosocial support groups and HIV care and treatment programmes.^28^ Additionally, governments should also work towards addressing policy barriers at the regional, national and subnational levels.^29^ McIntyre et al. added that social media and technology could be used to overcome these barriers and that there was an urgent need to reach young MSM in their spaces using the technologies and communication and/or media platforms they currently and mostly use.^30^

### Limitations

The experiences shared by participants in this study are limited to MSM in Mdantsane township and cannot be transferred to other townships in the Eastern Cape province.

A major unforeseen limitation of the study was the outbreak of coronavirus that affected the entire study plan, particularly the duration the primary researcher planned to complete the study. The timeframes and the entire project plan for the study had to be changed and adjusted to ensure that the study continued without compromising the health and well-being of the study participants and the quality and credibility of the study. Moreover, significant adjustments had to be implemented in line with the government coronavirus disease 2019 (COVID-19) Management Guidelines and the UKZN Research Ethics Guidelines to ensure the safety of all study subjects.

### Implications or recommendations

Men who have sex with men in this study expressed concerns about the unprofessional behaviour of healthcare workers, a lack of knowledge about MSM and poor client interactions. These issues not only violate MSM’s rights to respectful, non-discriminatory healthcare but also breach national guidelines, constituting unethical conduct. To address these problems, it is recommended that the government implement education and sensitisation training for healthcare workers, focussing on human sexuality, sexual orientation diversity and respectful, non-discriminatory conduct. Additionally, educating healthcare workers about MSM lifestyles and preferences is crucial for providing stigma-free, sensitive services. HIV Self-Testing (HIVST) is also recommended as a strategy to increase HCT uptake among MSM while minimising the risk of mistreatment by healthcare workers.^23,31^ In moderation, social scientists call for continuous efforts and interventions to de-stigmatise HIV and homosexuality locally and globally. These efforts should start by sensitising healthcare professionals about sexuality and diversity in sexualities.^24^

While the primary researcher believes that the above limitations did not in any way impact the primary objectives and outcomes of the study, future studies could be conducted, perhaps using a different theoretical framework that could better explore the experiences of MSM when seeking to access HCT services within their PHC centres.

## Conclusion

This study confirms that MSM often experience significant mistreatment from healthcare providers when seeking HCT and other services, leading to missed opportunities to know their HIV status and make informed sexual health decisions because of fear of marginalisation. Confidentiality also remains a serious challenge that MSM continue to experience when seeking HCT services from their local clinics. To address these issues, continuous efforts are necessary to de-stigmatise HIV and homosexuality through extensive sensitisation and sexual diversity education programmes for healthcare professionals in South Africa.
